# Gratitude promotes prosocial behavior even in uncertain situation

**DOI:** 10.1038/s41598-024-65460-z

**Published:** 2024-06-22

**Authors:** Ryuji Oguni, Chikara Ishii

**Affiliations:** 1 College of Comprehensive Psychology, Ritsumeikan University, 2-150 Iwakura-Cho, Ibaraki, Osaka Japan; 2 Center for Information and Neural Networks, Advanced ICT Research Institute, National Institute of Information and Communications Technology, and Osaka University, 588-2 Iwaoka, Iwaoka-Cho, Nishi-Ku, Kobe, Hyogo Japan

**Keywords:** Gratitude, Prosocial behavior, Uncertainty, Social mindfulness, SoMi paradigm, Psychology, Human behaviour

## Abstract

Gratitude is pivotal in promoting and maintaining prosocial interactions in human society. However, it is unclear whether the prosocial function of gratitude can be observed even in situations of uncertainty about whether one can provide benefits to others. Here, we examined whether gratitude promotes prosocial behavior in uncertain situations. Participants (N = 60) were randomly assigned to either a gratitude or neutral group. Following the emotion-induced manipulation, we examined whether participants would choose a non-unique resource when selecting one of four resources (one unique and three non-unique) to leave the choice to the follower. This represented an uncertain situation in which choosing a non-unique resource does not necessarily mean the follower will choose the unique one. Results showed that participants in the gratitude group were more likely to choose non-unique resources than those in the neutral group, suggesting that gratitude promotes prosocial behavior even in uncertain situations. Our findings indicate that gratitude is widely prevalent as a lubricant for interpersonal and cooperative relationships in human society.

Gratitude is a positive emotion individuals feel in receiving kindness from others^1^, and is pivotal in promoting and maintaining prosocial interactions^2,3^. Previous research has shown that gratitude promotes prosocial behavior, such as helping and sharing resources, in situations where it can reliably provide benefits to others^4–8^. These behaviors can reliably provide psychological benefits (e.g., satisfaction and happiness) and material benefits (e.g., money and goods) to the recipient. Does gratitude promote prosocial behavior in situations where it is uncertain whether such benefits can be provided to others? In everyday life, we often face situations in which we cannot reliably provide benefits to others because we do not always know the preferences and needs of others. In such situations, people may hesitate to act prosocially because the costs paid to provide benefits to others may be wasted. In this study, we aim to clarify whether the prosocial function of gratitude as a lubricant for interpersonal and cooperative relationships is widespread in human society by examining whether gratitude promotes prosocial behavior even in uncertain situations.

Gratitude motivates prosocial behavior^9^. It promotes helping and sharing behaviors, and its effects extend to not only to the benefactor but also to strangers^4–7^. The prosocial function of gratitude is triggered even when individuals cannot anticipate a return, such as the anonymous distribution of resources to others who require them^5^. Gratitude also reduces the risk of loss from interpersonal conflict by respecting the desires of others over one's own^10^. These studies examine the prosocial function of gratitude in situations where participants know the needs of others and can reliably provide psychological and material benefits to others. In everyday life, however, we also often decide whether to act prosocially in uncertain situations where we do not know whether we can reliably provide such benefits to others. This is because we do not always know the preferences and needs of others. To our knowledge, however, no studies have directly examined whether gratitude promotes prosocial behavior in uncertain situations. This suggests that the findings in the existing literature may not fully address the prosocial function of gratitude in a complex and dynamic human society. we seek to shed light on prosocial behavior in situations of uncertainty about whether one can reliably provide benefits to others, called social mindfulness (SoMi), and determine whether the prosocial function of gratitude is triggered even in uncertain situations.

SoMi is defined as thinking about others in the present and considering their needs and desires before decision-making^11,12^. It entails making considerate choices involving both the skill and the will to act in a way that is mindful of other people’s control over outcomes^11^. Imagine the following scenario. You enter a café and notice a slice of cheesecake and three chocolate cakes on display. If you order the chocolate cake, the person in line behind you can still order either the chocolate cake or the cheesecake. In other words, your behavior provides the person behind you a choice. However, even if you order the chocolate cake, it is uncertain whether you are providing a benefit to those behind you because the person behind you in line may also order the chocolate cake instead of the cheesecake. Thus, you can reliably leave a choice to provide a benefit to those behind you, but you cannot reliably provide psychological and material benefits to them. In this regard, SoMi is different from helping and sharing behaviors which can reliably provide such benefits to others^13^.

The SoMi paradigm is an established task for measuring SoMi. It requires participants to imagine performing a task with another participant and choosing a resource from one unique and three non-unique resources^14^. This paradigm does not present information about the other participant’s preferences and needs. In this scenario, choosing a non-unique resource is defined as prosocial behavior intended to provide benefits to others because it leads to leaving options for others^11,12^. The choice of non-unique resources also appears to be a self-oriented decision, such as the desire to obtain plentiful resources instead of prosocial behavior, but this possibility is ruled out by the finding that its choice is reduced in the absence of others^15^. Recent SoMi research has produced several findings from laboratory^11^, online^14^, and field^15^ experiments using the SoMi paradigm. For example, participants instructed to engage in other-oriented behavior are more likely to choose non-unique resources than those instructed to engage in self-oriented behavior^11^. The key to understanding the SoMi paradigm is the uncertainty about whether one’s actions will provide benefits to others. This represents the uncertain situation where choosing a non-unique resource does not necessarily mean that another participant will choose a unique resource.

This study examined whether gratitude promotes prosocial behavior in uncertain situations using the SoMi paradigm. Previous studies have shown that gratitude promotes prosocial behavior in situations in which individuals can reliably provide benefits to others^4–7^. Based on these findings, we hypothesized that gratitude would also promote prosocial behavior in uncertain situations. Specifically, we predicted that participants in the gratitude group would be more likely than participants in the neutral group to choose a non-unique resource so as to provide choices to others.

## Transparency and openness

We report our sample size, all data exclusions, all manipulations, and all measures in the study. All data have been made publicly available at the Open Science Framework (https://osf.io/bku87/). This study’s design and its analysis were not preregistered.

## Method

### Participants

The research design consisted of a between-participants, one-factor, two-level (gratitude group, control group) experiment. The sample size was determined prior to any data analysis. Using G*Power 3.1.9.4^16^, we conducted a preliminary power analysis based on the effect sizes (effect size *d* = 0.80, alpha level = 0.05, power = 0.80) reported in previous studies^5–7^. The effect size reported in related studies showing that gratitude promotes sharing behavior were larger than the nonabsolute but commonly used criterion for a large effect size (i.e., *d* > 0.80)^5–7^. Because we planned to apply a paradigm different from previous studies, we conservatively set the large effect size at *d* = 0.80. The results indicated that the minimum sample size for each group was 26 participants. We preliminarily determined to collect data from 30 participants in each group to account for the possibility of excluding data (e.g., equipment malfunction and experimenter error). We did not stop sampling at arbitrary points and collected data until the above number of participants was reached. Therefore, we collected data from 60 undergraduate students (18 males and 42 females; *M*_age_ = 19.20, *SD* = 2.83). We recruited only native Japanese speakers through the research participation system of the Faculty of Psychology (https://sona-systems.com). Participants were randomly assigned to either the gratitude (*n* = 30; 9 males, 21 females; *M*_age_ = 19.53, *SD* = 3.94) or the neutral group (*n* = 30; 9 males, 21 females; *M*_age_ = 18.87, *SD* = 0.73). Participants received course credit.

### Stimuli and apparatus

We used 12 image stimuli (https://www.socialmindfulness.nl/paradigm) identical to Van Doesum et al.^14^. We presented the stimuli on a 23.8-inch display (I-O DATA LCD-RDT241XPB, resolution 1920 × 1080 pixels) using E-Prime software (Version 3.0, Psychology Software Tools, Inc., Pittsburgh, PA, USA, https://pstnet.com/). The distance between the participant’s head and the display was approximately 60 cm.

### SoMi paradigm

This study followed the same procedure as Van Doesum et al.^14^. Participants were instructed to imagine a situation in which they had to choose one of several items with an imaginary stranger (a person they did not know and were unlikely to meet in the future). It was explained to participants that they would always choose the item first and that the item they chose could not be replaced. Participants then experienced two conditions: experimental and control. In the experimental condition, participants were presented with one unique item (e.g., one red cup) and three non-unique items (e.g., three green cups). In the control condition, they were presented with two identical items (e.g., two red cups and two green cups). A total of 24 trials were performed, with 12 image stimuli in each condition. Item placement was randomized within trials, and the order of the trials was randomized across conditions. The unique item type (e.g., red cup or green cup) was counterbalanced across participants.

### Procedure

This study was conducted individually in the university laboratory. First, we asked participants about their emotional state (gratitude, excitement, guilt, tension) at that moment (e.g., “Do you feel gratitude right now?”). Participants rated their emotional state on a 5-point scale ranging from 1 (not at all) to 5 (extremely). Participants then recalled an autobiographical memory for 5 minutes^17^. Participants in the gratitude group read the following instructions to recall an experience of gratitude. “Please recall carefully and in detail a specific experience in the past when you felt sincerely grateful for someone’s kindness or help.” Participants in the neutral group read the following instruction to recall a morning routine. “Please recall carefully and in detail the sequence of your morning routine (e.g., brushing teeth, changing clothes).” Participants then rated their emotional state again. In the above emotional state questions and emotion induction manipulations, we used the same instructions and items as in previous studies on the prosocial function of gratitude in Japanese populations^18,19^. The order of presentation of the four emotions (gratitude, excitement, indebtedness, and tension) in the emotion ratings before and after the emotion-induction manipulation was randomized between participants. The order was fixed within participants. Finally, the SoMi paradigm was conducted.

### Ethics statement

The study was approved by the Ritsumeikan University Ethics Review Committee for Research Involving Human Subjects (Kinugasa-Human-2022–96) and was conducted after obtaining informed consent from participants. All methods were performed in accordance with the relevant guidelines and regulations.

## Results

### Manipulation check

The means and standard errors for feelings of gratitude at each point in time are presented in Table 1. A two-way mixed-design analysis of variance (ANOVA) with a group (gratitude, neutral) and time (pre, post) as factors was conducted to determine whether the emotion-induction manipulations affected participants’ feelings of gratitude. The results showed that the main effect of the group, *F* (1, 58) = 12.30, *p* < 0.001, partial *η*^2^ = 0.17, the main effect of time, *F* (1, 58) = 26.24, *p* < 0.001, partial *η*^2^ = 0.31, and the group × time interaction, *F* (1, 58) = 63.31, *p* < 0.001, partial *η*^2^ = 0.52, were significant. A simple main effect test also showed that the post-test level of gratitude in the gratitude group was higher than that at pre-test, *F* (1, 29) = 64.44, *p* < 0.001, partial *η*^2^ = 0.69. In contrast, the post-test level of gratitude in the neutral group was lower than that at pre-test, *F* (1, 29) = 5.97, *p* = 0.02, partial *η*^2^ = 0.17. There was no significant difference in the level of gratitude between groups at the pre-test, *F* (1, 58) = 0.20, *p* = 0.65, partial *η*^2^ = 0.003. Conversely, the gratitude group’s post-test gratitude level was significantly higher than the gratitude score of the neutral group in post-test, *F* (1, 58) = 57.40, *p* < 0.001, partial *η*^2^ = 0.50.

**Table 1:** Means and standard errors of emotion ratings, SoMi choice, and SoMi RT for each group.

	Group			
	Gratitude		Neutral	
	*M*	*SE*	*M*	*SE*
Emotion rating				
Gratitude pre	2.67	0.25	2.83	0.27
Gratitude post	4.67	0.10	2.40	0.28
Excitement pre	2.03	0.18	2.10	0.20
Excitement post	2.27	0.21	1.47	0.13
Indebtedness pre	1.73	0.19	1.77	0.21
Indebtedness post	2.63	0.25	1.63	0.21
Tension pre	3.13	0.20	2.83	0.23
Tension post	2.13	0.20	1.97	0.21
SoMi choice				
Exp. ununique	8.60	0.59	6.70	0.48
SoMi RT				
Exp. Unique	3297.54	462.85	4148.10	417.95
Exp. ununique	4005.43	560.54	3782.87	376.66
Cont	3440.93	300.68	3388.80	249.54

A two-way mixed-design ANOVA with a group (gratitude, neutral) and time (pre, post) as factors was conducted to examine the effects of the emotion-induction manipulations on other emotions (excitement, guilt, tension). For excitement, the main effects of the group, *F* (1, 58) = 3.13, *p* = 0.08, partial *η*^2^ = 0.05, and time, *F* (1, 58) = 1.58, *p* = 0.21, partial *η*^2^ = 0.03, were not significant; however, the group × time interaction, *F* (1, 58) = 7.40, *p* = 0.01, partial *η*^2^ = 0.11, was significant. There was no significant difference between the pre- and post-test levels of excitement in the gratitude group, *F* (1, 29) = 0.77, *p* = 0.39, partial *η*^2^ = 0.03. In contrast, the level of excitement in the neutral group was lower at the post-test than at the pre-test, *F* (1, 29) = 12.94, *p* = 0.001, partial *η*^2^ = 0.31. There was no significant difference between groups in the pre-test level of excitement, *F* (1, 58) = 0.06, *p* = 0.81, partial *η*^2^ = 0.001. In contrast, the post-test level of excitement in the neutral group was significantly higher than in the gratitude group, *F* (1, 58) = 10.06, *p* = 0.002, partial *η*^2^ = 0.15. For indebtedness, the main effect of the group was not significant, *F* (1, 58) = 3.66, *p* = 0.06, partial *η*^2^ = 0.06; however, the main effect of time, *F* (1, 58) = 5.11, *p* = 0.03, partial *η*^2^ = 0.08, and the group × time interaction, *F* (1, 58) = 9.27, *p* = 0.003, partial *η*^2^ = 0.14, were significant. There was no significant difference between the pre- and post-test levels of indebtedness for the neutral group, *F* (1, 29) = 0.79, *p* = 0.38, partial *η*^2^ = 0.03, but the post-test level of indebtedness for the gratitude group was higher than the pre-test, *F* (1, 29) = 8.73, *p* = 0.01, partial *η*^2^ = 0.23. There was also no significant difference between the groups in the pre-test level of indebtedness, *F* (1, 58) = 0.01, *p* = 0.91, partial *η*^2^ = 0.0002, but the indebtedness level of the gratitude group at post-test was significantly higher than the neutral group, *F* (1, 58) = 9.46, *p* = 0.003, partial *η*^2^ = 0.14. For tension, the main effect of group, *F* (1, 58) = 0.84, *p* = 0.36, partial *η*^2^ = 0.01, and the group × time interaction, *F* (1, 58) = 0.19, *p* = 0.66, partial *η*^2^ = 0.003, were not significant; however, the main effect of time, *F* (1, 58) = 38.15, *p* < 0.001, partial *η*^2^ = 0.40, was significant. It is also noted that the pre-test level of tension was higher than the post-test level.

### Effect of gratitude on prosocial behavior

**Figure 1:**
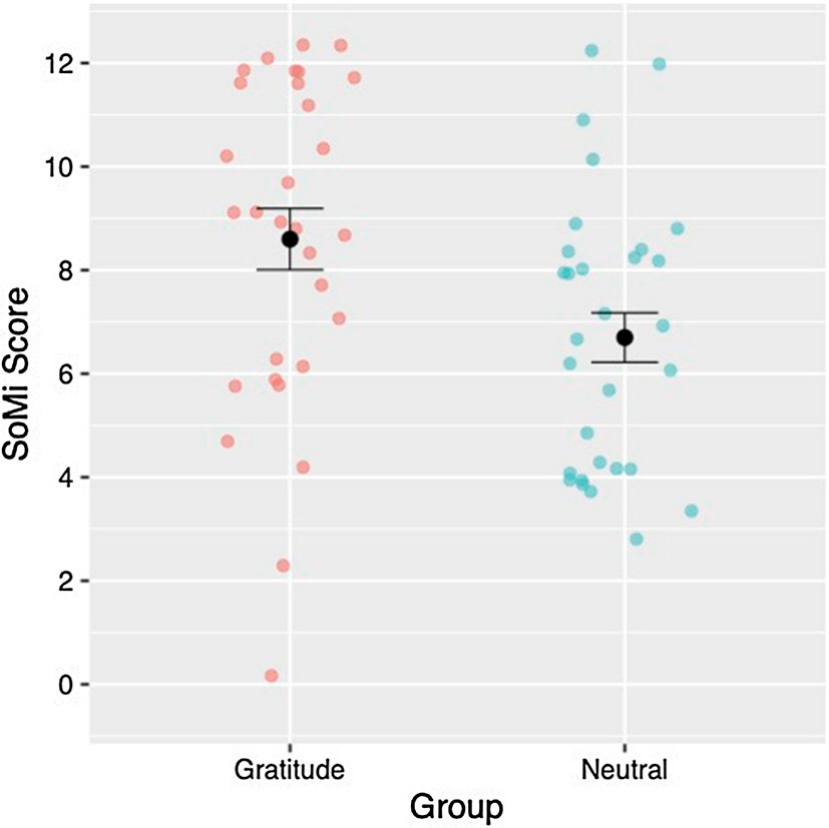
Black points and error bars indicate mean SoMi scores and standard errors for each group. Colored points represent individual SoMi scores.

We compared the item choice tendencies between the groups in the experimental condition. First, following Van Doesum et al. (2021)^14^, we scored the choice of unique items as 0 and the choice of non-unique items (i.e., SoMi) as 1; we then calculated the SoMi scores (ranging from 0 to 12; see Table 1 and Fig. 1). A Welch’s *t*-test revealed that the SoMi scores of the gratitude group were significantly higher than those of the neutral group, Welch’s *t* (55.50) = 2.50, *p* = 0.02, *d* = 0.64.

### Exploratory analysis

As an exploratory analysis, we compared the time taken to make choices between groups (see Table 1). First, we excluded 1 participant (gratitude group) who chose no non-unique item throughout the experimental conditions and 11 participants (gratitude group: 9, neutral group: 2) who chose no unique item. Welch’s *t*-tests indicated no significant differences between groups in the time taken to choose unique items, Welch’s *t* (45.75) = 1.36, *p* = 0.18, *d* = 0.38, and non-unique items, Welch’s *t* (44.11) = 0.33, *p* = 0.74, *d* = 0.09. Furthermore, there was no significant difference in response time between the gratitude and neutral groups in the control condition, Welch’s *t* (45.99) = 0.13, *p* = 0.89, *d* = 0.04.

We conducted an exploratory analysis of the effect of group on SoMi scores, including all post-test emotional scores (gratitude, excitement, guilt, tension) as a covariate. Results indicated that participants in the gratitude group were more likely to choose a non-unique resource than those in the neutral group, even when all post-test emotional scores were included as a covariate (*B* = 2.38, *t* (54) = 2.14, *p* = 0.04).

## Discussion

This study examined whether gratitude promotes prosocial behavior in uncertain situations. We conducted an emotion-induction manipulation and then used the SoMi paradigm to examine participants’ choice of one unique resource and three non-unique resources. The SoMi paradigm allowed for representing social contexts of high uncertainty, where the choice of a non-unique resource does not necessarily mean that followers would then choose the unique resource. We hypothesize that gratitude promotes prosocial behavior even in situations of uncertainty about whether one can provide benefits to others. Results indicated that participants in the gratitude group were more likely to choose a non-unique resource than participants in the neutral group, supporting the hypothesis. This indicates that the prosocial function of gratitude is triggered even in uncertain situations. This shows that gratitude promotes prosocial behavior even in uncertain situations. The present study adds to the literature on the prosocial function of gratitude, which has paid little attention to situational factors. Our findings suggest that the extent to which the prosocial function of gratitude is triggered is broader than previously thought.

Gratitude promotes prosocial behavior even in uncertain situations. Previous studies have examined this phenomenon only in situations where individuals can reliably provide psychological and material benefits to others^4–7^. However, we often face situations in which we are uncertain whether we can provide such benefits to others. In situations where the preferences and needs of others are unknown, people may hesitate to act prosocially to avoid wasting costs. However, the prosocial function of gratitude may be triggered even in uncertain situations where more consideration toward others is required. This study shows that gratitude increases the proportion of non-unique resource choices even in situations of uncertainty about whether leaving unique resources for others will benefit them. This shows that gratitude promotes prosocial behavior even in uncertain situations. Therefore, our findings suggest that the facilitating effect of gratitude on prosocial behavior is widely prevalent in human society than demonstrated in previous studies.

Our results suggest that the effects of gratitude on decision-making vary by context. Previous research has shown that gratitude inhibits risky decision-making for self-interest^20^. In contrast, the present study shows that gratitude facilitates risky decision-making for others-interest to act prosocially at the costs even in uncertain situations. This suggests that the effects of gratitude on decision making vary across contexts and provides a new perspective for research on their relationship.

Exploratory analyses showed no difference between groups in the time spent choosing unique and non-unique items. Although previous studies have used the time spent helping others and the number of resources distributed to others as indicators^4–7^, no studies have examined the time taken to engage in prosocial behavior. Our results suggest that it is less likely that participants in the gratitude group were more conflicted than participants in the neutral group when making these choices, and that the facilitation of prosocial decision-making by gratitude may have been based on relatively intuitive judgments. However, we excluded a large sample from the exploratory analysis. This requires further testing.

This study has some limitations. First, the followers are fictional. This limitation applies not only to our study but also to other studies using the SoMi paradigm^11^. However, we believe it is important to recognize this limitation because it has been pointed out that people’s hypotheses differ from their actual behavior^21^. Future research should examine whether grateful individuals act more prosocially toward real others under uncertainty in real situations using laboratory and field studies. Second, the effects of recalled content were not considered. In this study, participants in the gratitude group recalled social interaction experiences, whereas participants in the neutral group recalled personal experiences. Participants in the gratitude group may have reinforced their norms and beliefs that they should leave choices to others by recalling social interaction experiences^15,22^. Supporting this alternative explanation, additional exploratory analyses showed that participants in the gratitude group were more likely to choose a non-unique resource than those in the neutral group, even when post-test level of gratitude was included as a covariate. Note, however, that previous studies indicate that the facilitative effects of gratitude on prosocial behavior cannot be explained by simple social interactions^4–7^. Since our study compared only the gratitude and neutral groups, we cannot conclude whether the group difference in SoMi scores was due to recall of gratitude experiences or social interaction experiences. Future studies should rigorously test these possibilities by setting up groups to recall social interaction experiences that are not accompanied by gratitude experiences and by recording the content of the recall. Third, the emotion-induction manipulation also affected other emotions. Gratitude is a complex emotion that is predominantly positive but also mixed with negative emotions^23^. However, based on the amount of change and effect sizes both before and after the emotion-induction manipulation, the participants’ level of gratitude in the gratitude group indicated greater changes than the other emotions. This suggests that participants predominantly felt emotions derived from gratitude, not indebtedness. Future studies should examine whether these findings have a gratitude-specific effect by distinguishing between gratitude and indebtedness using a manipulation that emphasizes the benefactor’s good intentions^1^.

In conclusion, the present study demonstrates that gratitude promotes prosocial behavior even in uncertain situations. Our findings fill a gap in the literature and suggest that the prosocial function of gratitude is widely prevalent in human society. It also provides valuable insights into how gratitude builds and maintains good interpersonal and cooperative relationships in complex and dynamic societies characterized by uncertainty.

## Data Availability

The datasets generated during the current study are available in the Open Science Framework: https://osf.io/bku87/.
